# Modeling Tissue and Blood Gas Kinetics in Coastal and Offshore Common Bottlenose Dolphins, *Tursiops truncatus*

**DOI:** 10.3389/fphys.2018.00838

**Published:** 2018-07-17

**Authors:** Andreas Fahlman, Frants H. Jensen, Peter L. Tyack, Randall S. Wells

**Affiliations:** ^1^Global Diving Research, Ottawa, ON, Canada; ^2^Fundación Oceanografic de la Comunidad Valenciana, Valencia, Spain; ^3^Aarhus Institute of Advanced Studies, Aarhus University, Aarhus, Denmark; ^4^Sea Mammal Research Unit, Scottish Oceans Institute, University of St Andrews, St Andrews, United Kingdom; ^5^Chicago Zoological Society's Sarasota Dolphin Research Program, Mote Marine Laboratory, Sarasota, FL, United States

**Keywords:** diving physiology, modeling and simulations, gas exchange, marine mammals, decompression sickness, blood gases, hypoxia

## Abstract

Bottlenose dolphins (*Tursiops truncatus*) are highly versatile breath-holding predators that have adapted to a wide range of foraging niches from rivers and coastal ecosystems to deep-water oceanic habitats. Considerable research has been done to understand how bottlenose dolphins manage O_2_ during diving, but little information exists on other gases or how pressure affects gas exchange. Here we used a dynamic multi-compartment gas exchange model to estimate blood and tissue O_2_, CO_2_, and N_2_ from high-resolution dive records of two different common bottlenose dolphin ecotypes inhabiting shallow (Sarasota Bay) and deep (Bermuda) habitats. The objective was to compare potential physiological strategies used by the two populations to manage shallow and deep diving life styles. We informed the model using species-specific parameters for blood hematocrit, resting metabolic rate, and lung compliance. The model suggested that the known O_2_ stores were sufficient for Sarasota Bay dolphins to remain within the calculated aerobic dive limit (cADL), but insufficient for Bermuda dolphins that regularly exceeded their cADL. By adjusting the model to reflect the body composition of deep diving Bermuda dolphins, with elevated muscle mass, muscle myoglobin concentration and blood volume, the cADL increased beyond the longest dive duration, thus reflecting the necessary physiological and morphological changes to maintain their deep-diving life-style. The results indicate that cardiac output had to remain elevated during surface intervals for both ecotypes, and suggests that cardiac output has to remain elevated during shallow dives in-between deep dives to allow sufficient restoration of O_2_ stores for Bermuda dolphins. Our integrated modeling approach contradicts predictions from simple models, emphasizing the complex nature of physiological interactions between circulation, lung compression, and gas exchange.

## Introduction

The physiological adaptations that optimize foraging in marine mammals have long interested researchers. Optimal foraging theory implies that marine mammals should change dive behavior and metabolic pathways, and the fraction of aerobic and anaerobic metabolism based on dive depth and prey availability (Carbone and Houston, 1996; Cornick and Horning, 2003). Considerable work has been dedicated to understanding the aerobic limitations of diving air-breathing vertebrates, as these help with understanding foraging limits and efficiency. Kooyman et al. (1983) described the maximum dive duration until increasing blood lactate levels as the aerobic dive limit (ADL). The calculated ADL (cADL) was later defined as the total O_2_ stores divided by the rate of O_2_ consumption (Butler and Jones, 1997), and has been estimated in a number of species (Kooyman and Ponganis, 1998; Butler, 2006). However, metabolic rate may change over the course of a dive or foraging bout, and a few studies have subsequently estimated the cADL from measured (respirometry: Castellini et al., 1992; Reed et al., 1994, 2000; Hurley and Costa, 2001; Sparling and Fedak, 2004; Fahlman et al., 2008a, 2013) or estimated diving metabolic rate (doubly labeled water, or a proxy of metabolic rate: Boyd et al., 1995; Froget et al., 2001; Butler et al., 2004; Fahlman et al., 2008b).

Most studies agree that the majority of dive durations are well within the ADL/cADL, as this increases foraging efficiency and reduces lengthy surface intervals required to remove accumulated anaerobic by-products such as lactate (Kooyman et al., 1983). However, some species of otariids that feed on the benthos appear to exceed their cADL regularly, while those that feed in shallower water have shorter dive durations and seldom exceed the cADL (Costa et al., 2001). These differences may indicate true variation in foraging behavior, but may also be suggestive of morphological or physiological differences within or between closely related species that alter cADL (Hückstädt et al., 2016). For example, the muscle mass in previous studies was assumed to be similar to that of the Weddell seal (Costa et al., 2001), and such assumptions are often necessary as available data do not exist for all variables and species. However, variation in underwater swimming behavior (Williams, 2001; Fahlman et al., 2013), or morphological variation in muscle mass and fiber type (Pabst et al., 2016) may significantly alter the metabolic cost of foraging.

Large variations in dive behavior also exist within species. In the common bottlenose dolphin (*Tursiops truncatus*), different ecotypes have evolved to occupy different ecological niches (Mead and Potter, 1995; Hoelzel et al., 1998). Coastal bottlenose dolphins inhabit coastal areas and generally perform short (<60 s), shallow (<10 m) dives (Mate et al., 1995), while offshore bottlenose dolphins inhabit offshore, deep-water habitats and regularly dive below 200 m (Klatsky et al., 2007). Interestingly, neither resting metabolic rate nor lung compliance differed between the two ecotypes (Fahlman et al., 2018a,b), but blood hematocrit was significantly higher in the offshore ecotype (Klatsky et al., 2007; Schwacke et al., 2009; Fahlman et al., 2018a). In addition, the offshore ecotype is generally larger (Klatsky et al., 2007), possibly due to larger muscle mass to help increase the available O_2_ stores and diving capacity. In an attempt to better understand the physiological limitations of these two ecotypes, and to explore how variation in morphology and anatomy may alter gas dynamics during diving, we used a previously published gas exchange model (Fahlman et al., 2009; Hooker et al., 2009; Hodanbosi et al., 2016) to estimate blood and tissue gas tensions for O_2_ (PO_2_), CO_2_ (PCO_2_), and N_2_ (PN_2_) from high resolution (1 Hz) dive records from coastal bottlenose dolphins from Sarasota Bay, Florida, and offshore bottlenose dolphins sampled near the island of Bermuda.

## Materials and methods

### Model

The model described in this paper uses the breath-hold diving gas dynamics model developed by Fahlman et al. (2009) and Fahlman et al. (2006), which has been used to estimate lung, blood, and tissue gas tensions in a number of species (Hooker et al., 2009; Kvadsheim et al., 2012; Hodanbosi et al., 2016). A brief summary of the model is included below, with the specific changes made for the current modeling effort. The model was parameterized for bottlenose dolphins and was based on published values available for this species when possible, and otherwise on published values for beaked whales or phocids (as detailed below).

As in previous work, the body was partitioned into 4 compartments; brain (B), fat (F), muscle (M), and central circulation (CC), and one blood compartment (BL, arterial and mixed venous). In the current study, bone was included in the fat compartment as the bones of deep diving whales are high in fat content (Higgs et al., 2010). The central circulatory compartment included heart, kidney, and liver. The muscle compartment included muscle, skin, connective tissue, and all other tissues (Fahlman et al., 2009). In previous studies, the alimentary tract was placed in the central circulation. However, due to its lower metabolic rate, it was placed in the muscle compartment in the current study. The size of each compartment as well as the myoglobin and hemoglobin concentrations were initially taken from data on coastal dolphins (Mallette et al., 2016). As there is little or no information about the body composition of offshore dolphins, changes were made to the body composition of the offshore ecotype to be more like a beaked whale (Pabst et al., 2016).

### Lung gas stores and gas exchange

Gas exchange occurred between the lungs and blood compartment and between the blood compartment and each other compartment (Fahlman et al., 2006). The O_2_, CO_2_, and N_2_ stores in the lung consisted only of a gas phase and were assumed to be homogenous. We assumed that there was no diffusion resistance at the lung-surface interface when an animal was breathing at the water surface (Farhi, 1967). Thus, arterial blood tension of N_2_ (Pa_N2_), O_2_ (Pa_O2_), and CO_2_ (Pa_CO2_) were assumed to be equal to the alveolar partial pressures. For an animal breathing at the surface, we assumed that alveolar partial pressures of N_2_ (PA_N2_), O_2_ (PA_O2_), and CO_2_ (PA_CO2_) were, respectively, 0.74 ATA, 0.133 ATA, and 0.065 ATA (Fahlman et al., 2015), with 0.062 ATA being water vapor.

During diving, hydrostatic pressure compresses the respiratory system, which causes a pressure-dependent pulmonary shunt to develop (Fahlman et al., 2009). The parameters that describe the structural properties for the alveolar space and conducting airways (Equations 4 and 5 in Bostrom et al., 2008) were updated based on previously published compliance values for the bottlenose dolphin (Table 1, Fahlman et al., 2011, 2015; Moore et al., 2014).

**Table 1:** Parameters used to describe the structural properties of the respiratory system.

**Parameter**	**Equation**	
	**Equation 4**	**Equation 5**
*a*	1.05 ± 0.04 (1.1)	
*b*	0.90 ± 0.06 (1.2)	
*c*	2.51 ± 0.19 (1.3)	
*Kp*		−31.0 ± 23 (−12.8)
*n*		2.4 ± 1.4 (0.91)

*The parameters for Equations (4) and (5) in Bostrom et al. (2008) were revised using previously published compliance data from the bottlenose dolphin (Fahlman et al., 2018b). Old values are in parentheses*.

Total lung capacity (TLC) included the volume of the dead space (trachea and bronchi, V_D_), and the maximum alveolar volume (V_A_), i.e., TLC = V_D_ + V_A_. It was assumed that gas exchange only occurred in the alveoli and when the diving alveolar volume (DV_A_) was equal to 0, gas exchange stopped. We used the equation initially developed by Kooyman (1973), to estimate TLC (TLC_est_ = 0.135 $M_{\text{b}}^{0.92}$, where *M*_b_ is body mass in kg), and later validated for the dolphin (Fahlman et al., 2011, 2015). Dead space volume was assumed to be 7% of TLC (Kooyman, 1973; Fahlman et al., 2011). The relationship between pulmonary shunt and DV_A_ ${\text{V}}_{\text{A}}^{-1}$ was determined using a power function (Bostrom et al., 2008) for data from the harbor seals (Equation 6A in Fahlman et al., 2009).

All pressures were corrected for water vapor pressure, assuming that the respiratory system was fully saturated at 37°C.

### Blood and tissue gas stores

As in previous studies, the blood and tissue stores of N_2_, CO_2_, and O_2_ were determined by the solubility coefficients for each gas, as previously detailed (Fahlman et al., 2006). For O_2_ and CO_2_, the average concentration of hemoglobin for each population was used to estimate the blood O_2_ stores and CO_2_ storage capacity (Table 2). Tissue gas content was determined as previously detailed (Fahlman et al., 2006, 2009).

**Table 2:** Body compartment composition (% of body mass), estimated compartment metabolic rate (rest), and the value used in the model, cardiac output at rest/diving and at the surface, male adult dolphins.

**Tissue**	**Percent of body mass**		**References**
	**Sarasota**	**Bermuda**	
Blood	7.1	19.1	Ridgway and Johnston, 1966
Brain	1.0	0.2	Mallette et al., 2016
Fat	31.1	20.7	
Central circulation	3.73	3	
Muscle	57 (35)	57 (50)	
**Metabolic rate for a 200 kg animal (l O**_2_ **min**^−1^**)**			
Brain	0.035	0.011	Yazdi et al., 1999; Yeates and Houser, 2008; Noren et al., 2013; Fahlman et al., 2015, 2018a
Fat	0.039	0.024	
Central circulation	0.321	0.255	
Muscle	0.273	0.245	
$\overset{\cdot }{V}O_{2tot}$ rest	0.67$	0.54$	
${\overset{\cdot }{\text{Q}}}_{\text{tot}}$ rest (l min^−1^)	6.0¶	6.0¶	Miedler et al., 2015
**Variables for O**_2_ **storage**			
[Hb] (g/100 g)	16	16	Ridgway and Johnston, 1966; Noren et al., 2002; Hall et al., 2007; Schwacke et al., 2009
[Mb] (g Mb/100 g muscle)	3	7.3	Noren et al., 2001; Ponganis, 2011; Pabst et al., 2016
Hct (%)	44	57	Ridgway and Johnston, 1966; Hall et al., 2007; Schwacke et al., 2009
**O**_2_ **stores for a 200 kg dolphin (l)**			
Lung	2.21	2.21	Kooyman, 1989; Kooyman and Ponganis, 1998; Fahlman et al., 2015, 2017b
Tissue	3.82	10.00	
Blood	2.68	7.50	

*For muscle, the value in parenthesis is the actual muscle mass used with myoglobin. Data are for the resting data without the multiplier for exercise. $Values for active animals equal to 2x resting values. ¶Values for active animals equal to 3x resting values (Sarasota dolphins) or 7x resting values (Bermuda dolphins)*.

### Compartment size, cardiac output, and blood flow distribution

For the coastal ecotype sampled in Sarasota Bay, the relative size of each compartment was taken from previous studies in coastal bottlenose dolphins (Table 2; Ridgway and Johnston, 1966; Mallette et al., 2016). For blood and muscle we used species-specific values for blood hematocrit (Ridgway and Johnston, 1966; Hall et al., 2007; Schwacke et al., 2009; Fahlman et al., 2018b), and myoglobin concentration (Noren et al., 2001; Ponganis, 2011). For the deep-diving offshore dolphins, we initially assumed that the animals were similar to the coastal ecotype from Sarasota Bay. However, these assumptions resulted in a cADL that was too short for all Bermuda dolphins. We therefore adjusted the body composition of the Bermuda dolphins, assuming that their body composition was similar to that of deep diving beaked whales (Pabst et al., 2016), and used the body composition for *Mesoplodon* from our previous studies (Table 2; Hooker et al., 2009).

For the current study, we assumed that the cardiac output (${\overset{\cdot }{\text{Q}}}_{\text{tot}}$) at the surface was 3 times higher than the ${\overset{\cdot }{\text{Q}}}_{\text{tot}}$ measured in resting bottlenose dolphins (31.5 ml min^−1^ kg^−1^, Miedler et al., 2015). To account for differences in *M*_b_, mass specific ${\overset{\cdot }{\text{Q}}}_{\text{tot}}$ (s${\overset{\cdot }{\text{Q}}}_{\text{tot}}$) was adjusted using Equation (5) in Fahlman et al. (2009). During diving, the ${\overset{\cdot }{\text{Q}}}_{\text{tot}}$ was reduced to 1/3 of the value at the surface to account for the dive response, i.e., the diving ${\overset{\cdot }{\text{Q}}}_{\text{tot}}$ was the same as that measured in resting animals. Blood flow distributions to each tissue at the surface and during diving were iteratively tested to maximize utilization of O_2_ to increase the aerobic dive duration (Table 2).

### Dive data used

We used dive data collected from high resolution digital audio and movement recording tags (DTAG, Johnson and Tyack, 2003) attached to the dorsal side of each dolphin by means of four small suction cups, and programmed to release after <24 h. DTAGs were deployed during 2013–2016 in Sarasota Bay and in August of 2016 off the coast of Bermuda, during capture-release operations (e.g., Wells et al., 2004; Klatsky et al., 2007). The dolphins were tagged and data collected under permits issued by NMFS (Scientific Research Permit Number 15543) and the Bermuda Government, Department of Environment and Natural Resources (Research permit number SP160401r).

Data processing was done using the DTAG toolbox (soundtags.st-andrews.ac.uk). Tag depth was sampled at 200 Hz and subsequently down-sampled to 25 Hz during post-processing using a linear phase 10 Hz low-pass FIR filter. Surfacings were used to estimate the zero-pressure offset and to characterize and remove the effect of temperature on estimated depth.

A dive was defined as a submergence deeper than 1.5 m (1.15 ATA) and longer than 10 s. The start and end of each dive was calculated as the first and last point of the dive that exceeded 0.1 m depth, and the surface interval was defined as the time from current dive to previous dive.

## Results

### Respiratory compliance

**Figure 1:**
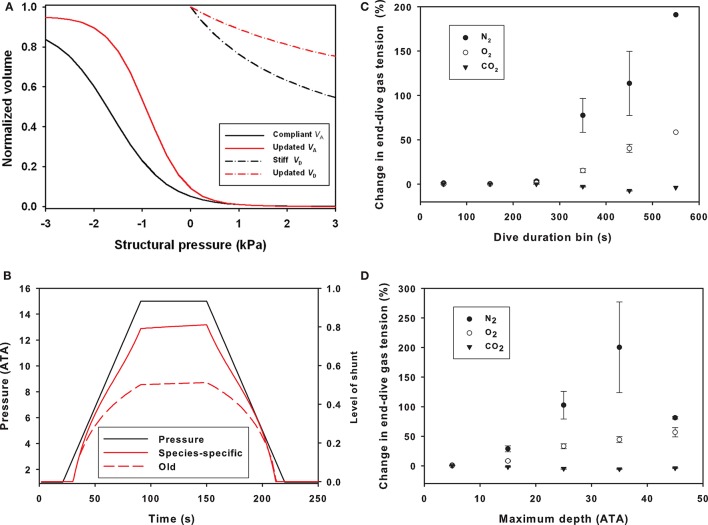
**(A)** Normalized volume (*V*_A_, alveolar volume; *V*_D_, dead space/tracheal volume) vs. structural pressure for alveolar and dead space compliances based on the estimate from Bostrom et al. (2008), or updated estimates from bottlenose dolphins (Fahlman et al., 2011, 2015). **(B)** Differences in pulmonary shunt with old and updated compliance values for the respiratory system in dolphins during a representative dive to 150 m for an animal with a body composition like the Bermuda dolphin (Fahlman et al., 2009). The average compliant alveoli and medium compliant trachea were used for the base model, and this model was used as a basis of comparison with all other simulations. Changes in end-dive mixed venous N_2_, O_2_, and CO_2_ levels against **(C)** dive duration (sec) or **(D)** maximum dive depth (ATA) when comparing old and revised lung compliance values. The y-axis is the change in percent for [(old-new)/old ^*^ 100]. These changes reflect how the structural properties alter the shunt and ventilation-perfusion mismatch (Garcia Párraga et al., 2018).

The updated parameters that defined the structural properties (compliance) of the respiratory system resulted in a stiffer upper airway and more compliant alveolar space as compared with the values used in previous studies (Figure 1A), which affected the pulmonary shunt (Figure 1B). The parameters for respiratory compliance were updated and the model output compared with the results from the previous model (Figures 1C,D). The end-dive gas tension increased with both dive duration and maximum dive depth for N_2_ and O_2_, while for CO_2_ there was a slight decrease (Figures 1C,D).

### Dive data

**Figure 2:**
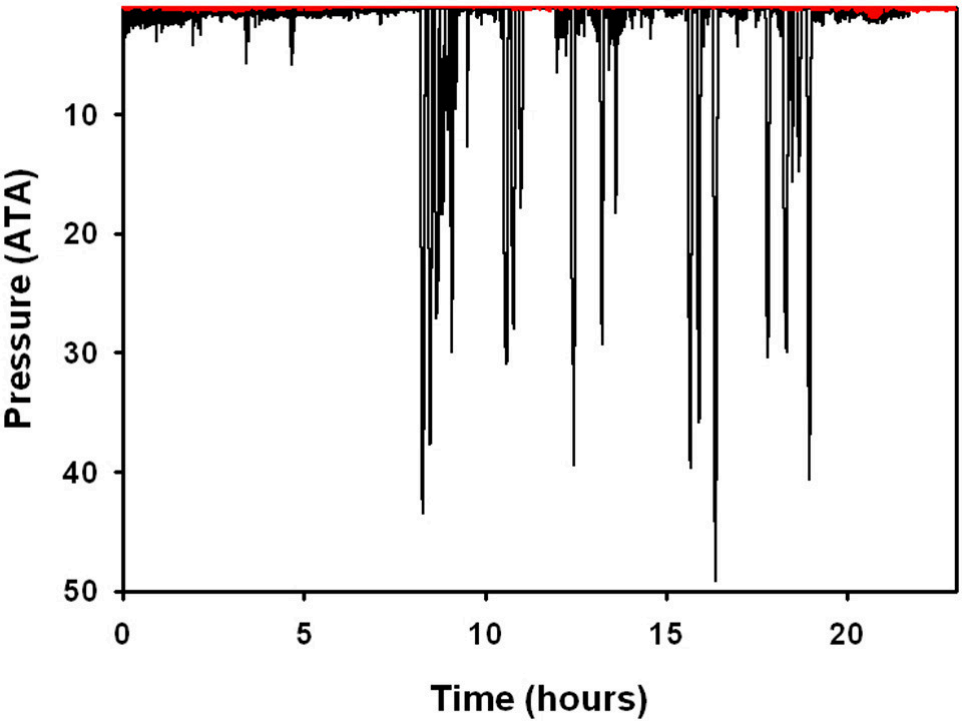
Representative dive data from two bottlenose dolphins in Sarasota Bay, Florida (red line), and Bermuda (black line). Data are plotted on the same axes range to show the differences in diving capacity/behavior. Pressure in ATA where 1 ATA is at the surface and 2 ATA is at 10 m depth.

**Figure 3:**
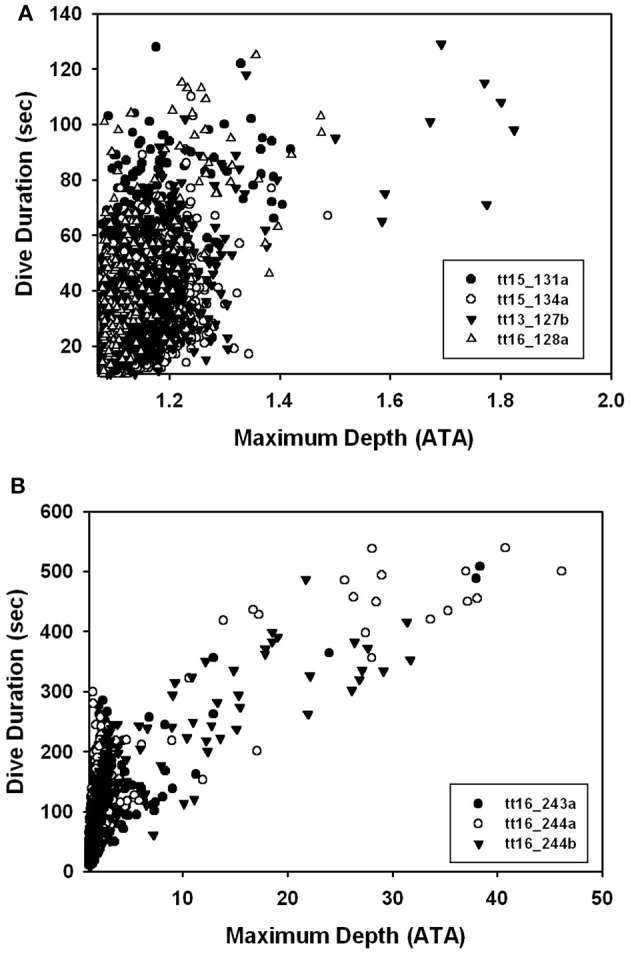
Dive depth vs. dive duration for **(A)** coastal Sarasota and **(B)** offshore Bermuda dolphins.

There were no significant differences in body mass (*M*_b_, *P* > 0.1, Welch *t*-value: 1.8, df = 2), body length (*P* > 0.9, *t*-value: 0.02, df = 5), average number of dives per hour (*P* < 0.1, Welch *t*-value: 0.3, df = 5), and average surface interval (*P* > 0.1, Welch *t*-value: 1.3, df = 2, Table 3) between ecotypes. There were significant differences in the dive behavior between the two populations, with dive duration (*P* < 0.01, Welch *t*-value: 4.37, df = 2), maximum dive depth (*P* < 0.05, Welch *t*-value: 3.95, df = 2), and mean depth per dive (*P* < 0.05, Welch *t*-value: 3.57, df = 2) being significantly higher in the Bermuda dolphins (Table 3, Figures 2, 3). For dives < 60 s, the Sarasota dolphins never exceeded a dive depth of 5 m (Figure 3A). As the dive duration increased >60 s, so did the maximum depth and also the variation (Figure 3A). For the Bermuda dolphins, there was a significant increase in the dive duration as the dive depth increased (Figure 3B). Dives < 60 s showed little variation and few exceeded 10 m (Figure 3B). As the dive duration increased beyond 100 s, the variation in maximum dive depth increased and dives exceeding 100 m became more common (Figure 3B). We found no indication that the surface interval increased with dive duration of the previous (*X*^2^ = 0.27, df = 1, *P* > 0.6) or next dive *X*^2^ = 0.05, df = 1, *P* > 0.8).

**Table 3:** Descriptive metrics for animals in study.

**Animal ID**	**Place**	**Sex**	***M*_b_**	**Age**	**Length (cm)**	**No. dives**	**Dives per hour**	**DD (s)**	**Maximum dive depth (m)**	**Mean depth (m)**	**Surf interval (s)**	**No. dives >100 m**	**No. dives >200 m**
tt13_127b (FB90)	S	F	198	43	259	522	23	35 ± 20 (10-129)	2 ± 0 (1–10)	2 ± 0 (1–8)	124 ± 743 (1–1,4956)	0	0
tt15_131a (FB123)	S	F	166	17	241	1032	50	36 ± 19 (10–128)	2 ± 0 (1–5)	1 ± 0 (1–4)	36 ± 128 (1–2,015)	0	0
tt15_134a (FB199)	S	F	142	13	236	684	39	28 ± 16 (10–110)	2 ± 0 (2–6)	1 ± 0 (1–4.1)	65 ± 165 (1–3,340)	0	0
tt16_128a (FB33)	S	F	195	34	258	214	9	46 ± 25 (10–125)	2 ± 0 (2–6)	1 ± 0 (1–5)	363 ± 2,165 (1–22,572)	0	0
Average			175 ± 26	27 ± 14	248 ± 12	613 ± 341	30 ± 18	36 ± 7^*^	2 ± 0^*^	1 ± 0^*^	147 ± 149		
tt16_243a (Tt0019)	B	M	294	NA	256	491	28.1	65 ± 68 (10–508)	10 ± 21 (1–309)	6 ± 8 (1–247)	62 ± 85(1–706)	6	3
tt16_244a (Tt0021)	B	F	173	NA	238	805	37	63 ± 81 (10–539)	12 ± 37 (2–482)	8 ± 18(01–280)	34 ± 86 (1–1,508)	7	4
tt16_244b (Tt0022)	B	M	282	NA	251	513	30	82 ± 86 (10–487)	20 ± 38 (2–328)	12 ± 17 (1–176)	37 ± 68 (1–1,161)	30	11
Average			250 ± 67		248 ± 9	603 ± 175	32 ± 5	70 ± 10^*^	14 ± 5^*^	9 ± 3^*^	44 ± 15		

Animal dataset (and ID code), dolphin population (Sarasota-S, Bermuda-B), sex (F-female, M-male), body mass (M_b_, kg), straight length, average (± SD) number of dives (No. dives), average dives per hour, average dive duration (sec), average maximum dive depth, average mean depth, and average previous surface interval.

* *Significant difference between Sarasota and Bermuda animals*.

### Cardiac output and blood flow distribution

For both populations, we assumed that the diving ${\overset{\cdot }{\text{Q}}}_{\text{tot}}$ was equal to resting values measured in the bottlenose dolphins (Table 4; Miedler et al., 2015). The blood flow distribution for the Sarasota dolphins was able to vary greatly, due to their shorter and shallower dive pattern. For the Bermuda dolphins, deviation from a certain blood flow distribution caused tissues to run out of O_2_. The specific variation varied slightly between individual animals, but one distribution pattern that focused perfusion to the central circulation, and minimized flow to the muscle, allowed all dolphins to complete their dives aerobically (Table 4).

**Table 4:** Blood flow distribution (% of total cardiac output), cardiac output (${\overset{\cdot }{\text{Q}}}_{\text{tot}}$), rate of O_2_ consumption, for central circulation (CC), muscle (M), brain (B), and fat (F) body compartments for a 200 kg dolphin for animals in Sarasota or Bermuda.

**Location**	**State**	**Tissue**				**${\overset{\cdot }{\text{Q}}}_{\text{tot}}$**	**$\overset{\cdot }{V}O_{2}$**

		**CC**	**M**	**B**	**F**	**(l** · **s**^−1^**)**	**(l O**_2_ · **min**^−1^**)**
Sarasota	Surface	25	65	7	3	0.301	1.34
Sarasota	Dive	70	17	7	6	0.100	1.34
Bermuda	Surface	60	34	4	2	0.704	1.08
Bermuda	Dive	81	9	6	4	0.100	1.08

*The ${\overset{\cdot }{\text{Q}}}_{tot}$ was assumed to be resting during diving and 3 times resting while at the surface for the Sarasota dolphins, but 7 times resting for the Bermuda dolphins. The $\overset{\cdot }{\text{V}}O_{2}$ was assumed to be the same at the surface and while diving and estimated to be 2 × the resting metabolic rate (Table 1)*.

The blood flow required at the surface to assure that tissues received enough blood to restore O_2_ stores at the surface differed between the two ecotypes. For the Sarasota dolphins, the minimum ${\overset{\cdot }{\text{Q}}}_{\text{tot}}$ at the surface was 3 times higher than during diving. A surface ${\overset{\cdot }{\text{Q}}}_{\text{tot}}$ at least 7 times higher than during diving was required for the Bermuda population to ensure that tissues were saturated with O_2_; any lower surface ${\overset{\cdot }{\text{Q}}}_{\text{tot}}$ would result in tissue PO_2_ continuously decreasing with each dive. In addition, for the Bermuda dolphins, changes in perfusion associated with diving, i.e., the dive response, was set to occur only for dives deeper than 20 m. Consequently, the elevated ${\overset{\cdot }{\text{Q}}}_{\text{tot}}$ was required at the surface to help restore blood and tissue O_2_ stores.

### Blood and tissue gas tensions

**Figure 4:**
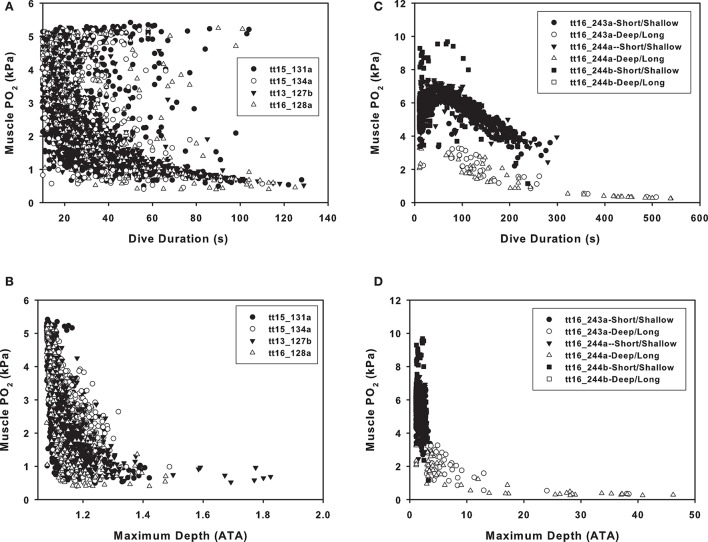
Estimated end-dive muscle PO_2_ (kPa) vs. **(A,C)** dive duration or **(B,D)** maximum dive depth (ATA, 1 ATA = 98.07 kPa) in **(A,B)** Sarasota or **(B,D)** Bermuda dolphins.

In the Sarasota dolphins, there was considerable variation in end-dive blood and tissue PO_2_ with dive duration. In Figure 4, the muscle is used as a representative tissue to show these variations. In Figure 4A, the large variation in muscle PO_2_ is shown when the muscle tension is plotted against dive duration. There was a consistent exponential decrease with dive depth (Figure 4B). A decrease in the blood and tissue PO_2_ was obvious in the Bermuda dolphins for both dive duration (Figure 4C) and depth (Figure 4D). For the Bermuda dolphins, the decrease in tissue PO_2_ varied depending on the level of pulmonary shunt, and the shunt decreased the end dive PO_2_ for dives of the same duration or maximum depth (Figures 4C,D). The effect of the shunt on end-dive PO_2_ was most obvious for the muscle compartment, but was also seen in the other tissues (data not shown).

**Figure 5:**
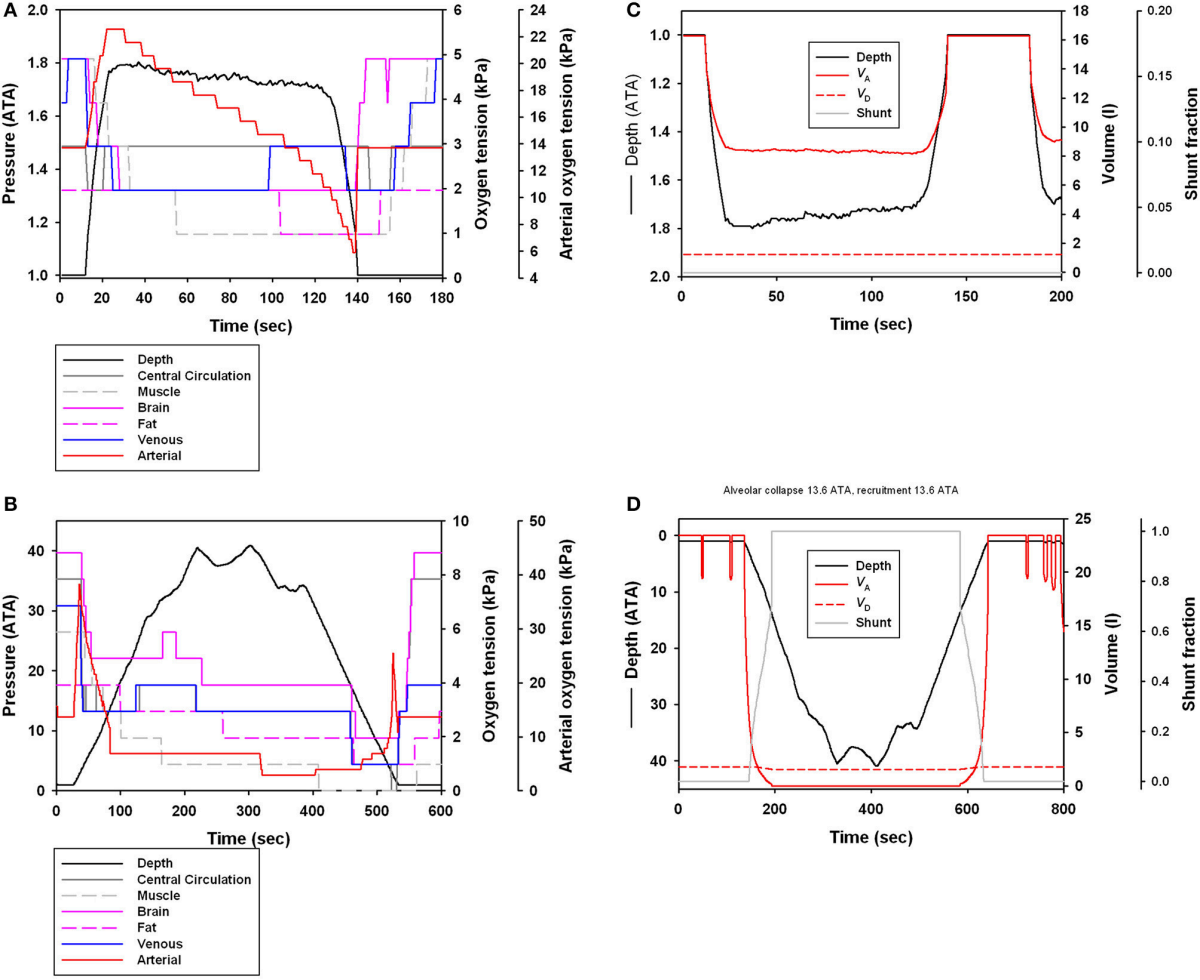
**(A,B)** Estimated central circulation, muscle, brain, fat, arterial, and venous PO_2_ (kPa) or **(C,D)** estimated shunt fraction, alveolar and tracheal volume for a long duration dive (depth in ATA, 1 ATA = 98.07 kPa) in **(A,C)** Sarasota and **(B,D)** Bermuda dolphin.

The estimated blood and tissue PO_2_ for a representative dive for dolphins from Sarasota (Figure 5A) or Bermuda (Figure 5B) showed similar patterns of a decrease in PO_2_ during a dive. However, the longer and deeper dive duration resulted in greater changes in blood and tissue PO_2_ values in the offshore ecotype. Figures 5C,D show the effect of depth on lung volume and pulmonary shunt.

## Discussion

In the current study, we modeled tissue and blood PO_2_, PCO_2_, and PN_2_ from fine-scale empirical dive data from bottlenose dolphins of both the coastal and offshore ecotypes to assess potential morphological or physiological adaptations that could help explain the large variation in dive behavior in these divergent populations. The results shows that the structural properties of the respiratory system have a significant effect on pulmonary gas exchange, and these changes are different for gases with different gas solubilities, agreeing with past work suggesting that variation in ventilation and perfusion may be important for managing gases during diving (West, 1962; Farhi and Yokoyama, 1967; Hodanbosi et al., 2016; Garcia Párraga et al., 2018). Furthermore, the results suggest that the deeper and longer dives of the offshore dolphins most likely reflect a greater O_2_ storage capacity, potentially combined with foraging of lower energetic cost. Future tagging studies to assess the energetic requirements of the different foraging strategies will be crucial to assess how close to their physiological limits each of these populations are living.

Theoretical work has suggested that the level of gas exchange, ${\overset{\cdot }{\text{Q}}}_{\text{tot}}$ and blood flow distribution are important to alter blood and tissue gas levels (Fahlman et al., 2006, 2009). Previously, we suggested that the structural properties of the respiratory system could have a significant effect on the level of gas exchange during breath-hold diving (Bostrom et al., 2008; Fahlman et al., 2009), and how man-made disturbances may alter the risk of gas emboli through changes in the dive profile or physiology (Hooker et al., 2009; Kvadsheim et al., 2012). However, species-specific estimates for the structural properties of the respiratory system were not available and published values from a range of species were used (Bostrom et al., 2008; Fahlman et al., 2009, 2014; Hooker et al., 2009; Kvadsheim et al., 2012), but recent work has suggested that species-specific estimates may significantly alter the model estimates (Hodanbosi et al., 2016). We therefore used recently published data for respiratory compliance (Fahlman et al., 2011, 2015, 2018b), ${\overset{\cdot }{\text{Q}}}_{\text{tot}}$ (Miedler et al., 2015), and metabolic rate (Fahlman et al., 2015, 2018a) for coastal and offshore bottlenose dolphin to update the model parameters. These changes altered the compression of the alveolar space, which affects the pressure dependence of the pulmonary shunt (Figure 1).

The updated parameters (Table 1), with stiffer airways and more compliant alveolar space, increased the level of shunt and reduced gas exchange throughout dives (Figures 1A,B). This significantly reduced N_2_ exchange during deeper dives as the alveolar space began compressing (Figures 1C,D). The updated parameters also affected O_2_ exchange, but the effect was less apparent. For CO_2_ there was little effect, and for longer and deeper dives, CO_2_ exchange was improved. This implies that variation in gas exchange from changes in the alveolar ventilation (${\overset{\cdot }{V}}_{A}$) and ${\overset{\cdot }{\text{Q}}}_{\text{tot}}$ relationship (${\overset{\cdot }{V}}_{A}$/${\overset{\cdot }{\text{Q}}}_{\text{tot}}$) differs for gases with varying gas solubility (West, 1962; Farhi and Yokoyama, 1967). These results agree with theoretical modeling in California sea lions that showed that changes in the structural properties of the respiratory system have a significant effect on the exchange of O_2_, CO_2_, and N_2_ that differs between gas species (Hodanbosi et al., 2016). Together these studies provide additional support for a recent hypothesis suggesting that the lung architecture and varying ${\overset{\cdot }{V}}_{A}$/${\overset{\cdot }{\text{Q}}}_{\text{tot}}$ would enable marine mammals to manipulate which gases are exchanged during diving (Garcia Párraga et al., 2018). While the current model does not include the proposed mechanisms that would alter the ${\overset{\cdot }{V}}_{A}$/${\overset{\cdot }{\text{Q}}}_{\text{tot}}$ relationship, e.g., the effect of heterogeneous pulmonary blood flow and alveolar compression (collateral ventilation), it shows that varying the structural properties, which effectively reduces the ${\overset{\cdot }{V}}_{A}$/${\overset{\cdot }{\text{Q}}}_{\text{tot}}$, the less soluble (N_2_) is significantly more affected as compared with the more soluble gases (O_2_ and CO_2_) (West, 1962; Farhi and Yokoyama, 1967). Thus, increasing upper airway stiffness and making the alveolar space more compliant causes the compression to occur at shallower depth, as originally hypothesized by Scholander (1940). This increases the level of shunt and reduces diffusion/ventilation, which has the greatest effect on gas with low solubility (West, 1962; Farhi and Yokoyama, 1967). In summary, we propose that the data in the current study agree with the suggestion that varying the ${\overset{\cdot }{V}}_{A}$/${\overset{\cdot }{\text{Q}}}_{\text{tot}}$ ratio may be an efficient way for deep divers to minimize N_2_ while still accessing pulmonary O_2_ and CO_2_ (Garcia Párraga et al., 2018).

The dive behaviors for the two dolphin forms were strikingly different, and demonstrate the large physiological plasticity in cetacean ecotypes. While these differences in dive behavior are undoubtedly related to differences in morphology, physiology and environment, the data presented here provide an interesting comparison of a species' capacity to vary physiological traits. Initially we used the available information for coastal dolphins to model tissue and blood gas dynamics in both populations (Table 2). For a 200 kg dolphin, the estimated O_2_ stores were 8.7 l (43.6 ml O_2_ kg^−1^), resulting in a calculated aerobic dive limit (cADL) of 6.5 min when assuming a field metabolic rate that is twice that of resting. This cADL is considerably greater than the observed maximum dive durations in the Sarasota dolphins ranging from 2.2 min in the current study (Table 3), or up to 4.5 min in previous work (R. Wells, unpublished observation, Wells et al., 2013). The diet of bottlenose dolphins in Sarasota is composed exclusively of fish (>15 different species found in stomach content of 16 stranded animals; Barros and Wells, 1998). Thus, their activity may require a variety of high energy activities from rapid body turns (pinwheel feeding) to tail slaps (fish whacking, kerpluncking) associated with prey capture (Nowacek, 2002), which would increase the daily metabolic rate and reduce the cADL. For a cADL of 2.2–4.5 min, the Sarasota ecotype would have a metabolic scope around 3-6 times their resting metabolic rate (3 times the estimated field metabolic rate in Table 3). The metabolic scope is the maximal aerobic metabolic rate divided by the basal metabolic rate and is typically in the range of 3–10; a scope >7 cannot be sustained over long periods (Peterson et al., 1990). Thus, a field metabolic rate that is 6 times higher than the resting value implies that this ecotype would be working at or close to the maximal aerobic capacity for a cADL of 2.2 min. However, another likely explanation is that the coastal dolphins here operate in an environment where they are seldom limited by their dive physiology. The bottlenose dolphins from which dive data were obtained primarily occupy shallow waters in and around Sarasota and Palma Sola bays, with water depths generally <10 m, and often less than a few meters. Thus, our results suggest that they can significantly increase their dive duration if necessary but that they may not need this to exploit prey in their shallow-water habitat.

The Bermuda dolphins, on the other hand, had significantly longer dives than the Sarasota animals. A considerable number of dives exceeded the cADL, even when calculated from twice the resting metabolic rate, and the dolphin ran out of O_2_ during long dives. For this reason, we hypothesized that the offshore dolphins must have increased O_2_ stores to help increase their dive duration. We are not aware of any published body composition data for offshore delphinids, and to increase the O_2_ stores we therefore modeled the body compartments according to the proposed body composition for deep-diving beaked whales, with increased blood and muscle O_2_ storage (Peterson et al., 1990) that significantly increased the total O_2_ stores (98.6 ml O_2_ kg^−1^), and the cADL (18.2 min, Table 2). With a maximal dive duration of 9 min, this provided a considerable scope for the Bermuda dolphins. The O_2_ storage capacity of the sperm whale (81 ml O_2_ kg^−1^), hooded seal (90 ml O_2_ kg^−1^), elephant seal (94 ml O_2_ kg^−1^), and Weddell seal (89 ml O2 kg-1, Ponganis, 2015) are lower than our estimated value. The calculations made in the current study are based on assumptions about the blood volume, and muscle mass of these animals, and it is likely that the O_2_ storage capacity is lower and the cADL shorter. If we assume that the longest dive represent the upper limit of the cADL, we would need an O_2_ storage capacity of approximately 49 ml O_2_ kg^−1^). Thus, we can predict that the O_2_ storage capacity is somewhere between these two values for the offshore ecotype.

In addition to a greater O_2_ storage capacity, Bermuda dolphins may also have a greater capacity to alter diving metabolic rate. In a previous study, we estimated the field metabolic rate of coastal ecotype bottlenose dolphins to be around 11.7–23.4 ml O_2_ min^−1^ kg^−1^. As there were no differences in the resting metabolic rate of the coastal (Fahlman et al., 2018a) or offshore populations (Fahlman et al., 2018b), this field metabolic rate for offshore dolphins resulted in a cADL of 4.2–8.4 min, which is closer to the maximum dive duration seen for these animals. Studies have indicated that the metabolic cost during longer and deeper dives is similar to or lower than the resting metabolic rate at the surface (Hurley and Costa, 2001; Fahlman et al., 2013), and deeper dives may provide cost savings as the animals may be able to glide during long portions of the dive (Hurley and Costa, 2001; Williams, 2001; Fahlman et al., 2013). Thus, offshore dolphins may also have reduced cost of foraging that increases their cADL. Assessing field metabolic rate from these two populations using validated metabolic proxies, such as activity (acceleration, Fahlman et al., 2008b, 2013), or heart rate (McPhee et al., 2003), could be used to determine how energy is partitioned in this population and resolve some of these questions. In fact, the DTAG data provide such an opportunity and is an objective for future studies.

The dive data from the Bermuda dolphins are suggestive of 2 different types of dives; one shallow (10–70 m, Figure 3B) that changes little in depth with duration and another dive type starting at around 100 m depth with a steep increase in dive duration with depth (Figure 3B). It is not surprising that deeper dives are generally longer, as the transit to depth is a significant portion of the dive duration. During the deeper dives, the pulmonary shunt alters the blood and tissue gas content, as shown for the end-dive muscle PO_2_ in Figure 4C. Without access to pulmonary PO_2_, the blood O_2_ was reduced and more O_2_ used from the muscle, pushing down end-dive PO_2_. This was not seen in the shallow diving Sarasota dolphins (Figure 4A), which never dove to depths where the pulmonary shunt began to alter gas tensions.

The differences in dive behavior had a significant effect on the gas tension in the blood and tissues (Figure 5). For both populations there was a continuous decrease in blood gases during the dive but the decrease was more extreme for the Bermuda dolphins (Figure 5B), and matching the blood flow to various tissues was more restrictive. This agrees with the suggestion made in previous modeling work that an important aspect of the dive response is to distribute the available perfusion to central tissues like the heart and brain, while blood flow to the muscle should be minimal (Davis and Kanatous, 1999). This allows muscle metabolism to be fueled by endogenous O_2_ and assures that the matching of utilization of endogenous and vascular O_2_ increases the duration of the dive that is fueled by aerobic metabolism (Davis and Kanatous, 1999).

For both ecotypes, the ${\overset{\cdot }{\text{Q}}}_{\text{tot}}$ while diving was assumed equal to the resting value measured in bottlenose dolphins (Miedler et al., 2015). The surface ${\overset{\cdot }{\text{Q}}}_{\text{tot}}$ in the Sarasota ecotype was set to 3 times higher than resting, which sufficiently restored O_2_ and removed CO_2_ from the blood and tissues to avoid continuous changes with repeated dives. In the Bermuda animals, on the other hand, a surface ${\overset{\cdot }{\text{Q}}}_{\text{tot}}$ of 3 times resting was not sufficient to restore the O_2_ or remove the CO_2_ during the longer dives. Initially, ${\overset{\cdot }{\text{Q}}}_{\text{tot}}$ was increased to 7 times higher than during diving, but while this improved restoration of blood and tissue O_2_ and CO_2_ levels, there were still continuous changes with repeated dives. By keeping the ${\overset{\cdot }{\text{Q}}}_{\text{tot}}$ elevated for submersions <20 m, however, we were able to sufficiently restore O_2_ and remove enough CO_2_ to prevent accumulating changes across repeated dives. These results suggest that ${\overset{\cdot }{\text{Q}}}_{\text{tot}}$ needs to be maintained during intervening short and shallow dives to allow restoration of normal blood and tissue gas tensions. Staying submerged during this time may help increase the PO_2_ and thereby the uptake of O_2_. The suggestion that there is little or no cardiovascular modification during shallow dives may be controversial, as most studies have reported changes in heart rate during diving. For example, in the bottlenose dolphin resting at the surface or while submerged the average heart rate were 105 and 40 beats min^−1^, respectively (Noren et al., 2012). However, the past study provides an estimated surface resting heart rate that is influenced by the respiratory sinus arrhythmia (RSA), which will significantly elevate the resting values. In a more recent paper the surface resting heart rate when accounting for the RSA ranged from 27 to 54 beats min^−1^ (Miedler et al., 2015), thus not very different from the resting diving heart rate reported by Noren et al. (2012). In addition, in the past study it was also reported that the diving heart rate increased with underwater activity by between 40 and 79% (Noren et al., 2012). While our estimated ${\overset{\cdot }{\text{Q}}}_{\text{tot}}$ at depths shallower than 20 m may be overestimated, there is experimental evidence that the perfusion is modulated while submerged and future studies could look at how this changes during short and shallow dives.

In the Sarasota dolphins, the dive depth caused a reduction in alveolar volume, but the low pressure did not cause tracheal compression or induce a pulmonary shunt (Figure 5C). In the Bermuda dolphins, the arterial PO_2_ increased during the descent to a maximum value, and then rapidly declined until the alveoli were reinflated during the ascent at which a second peak was observed (Figure 5B). We previously suggested that this pattern of arterial gas tension would support Scholander's hypothesis of pressure-induced hyperoxia as the lungs are compressed, followed by an increasing shunt as the alveoli are compressed until atelectasis (alveolar collapse), in this case at 126 m (Figure 5D), when the arterial side reflects the mixed venous (Fahlman et al., 2009; McDonald and Ponganis, 2012). This is supported by empirically measured arterial and venous gas tensions in both seals (Falke et al., 1985) and sea lions (McDonald and Ponganis, 2012). As the dolphin ascends, the alveoli are recruited at a depth of 126 m and gas exchange commences again. At this point, there is a second peak as pulmonary O_2_ again saturates the blood (Figures 5C,D). The second peak for arterial PO_2_ is lower as compared with the peak right before atelectasis, despite equivalent pulmonary PO_2_. During the dive, the O_2_ in the blood is continuously consumed, causing both the arterial and venous PO_2_ to decrease. As the alveoli are recruited at depth, the pulmonary PO_2_ increases. The high pulmonary and low venous PO_2_ result in an elevated partial pressure gradient that favors diffusion and gas exchange. Thus, pulmonary O_2_ diffuses into the pulmonary capillary and helps saturate the arterial blood. In addition, CO_2_ is removed from the blood into the lung. Both these processes help prepare the dolphin to minimize the duration of the surface interval as CO_2_ is being removed and O_2_ being taken up before reaching the surface. Elevated gas exchange at depth also increases N_2_ exchange, increasing the tissue and blood tension and the risk for gas emboli. However, the elevated pulmonary pressure as the alveoli open results in an elevated ${\overset{\cdot }{V}}_{A}$/${\overset{\cdot }{\text{Q}}}_{\text{tot}}$ ratio, favoring O_2_ and CO_2_ exchange, while limiting N_2_ exchange (Garcia Párraga et al., 2018). In addition, it has been hypothesized that marine mammals are able to separate the lung into two regions; one region that is ventilated and another atelactic area, with the latter being perfused (Garcia Párraga et al., 2018). This matching of pulmonary blood flow to hypoxic/atelactic regions result in selective gas exchange (Olson et al., 2010; Garcia Párraga et al., 2018), which during normal dives would help reduce the risk for gas emboli, and help prepare the dolphin to restore the O_2_ used and removing the CO_2_ produced (West, 1962; Farhi and Yokoyama, 1967; Olson et al., 2010). There is also evidence for an arterio-venous shunt that alters the blood gas tensions and could help minimize inert gas uptake during the ascent and help arterialize venous blood (Garcia Párraga et al., 2018).

In a number of marine mammals, there is an anticipatory pre-surfacing tachycardia, which likely increases ${\overset{\cdot }{\text{Q}}}_{\text{tot}}$ (Fedak et al., 1988; Thompson and Fedak, 1993; Andrews et al., 1997; Noren et al., 2012; Williams et al., 2017). During natural undisturbed dives, it seems that the change in heart rate begins during the initial stages of the ascent, but a large increase occurs during the later stages of the ascent close to the surface. Close to the surface, the blood and tissue tension would be supersaturated with N_2_ and the gas would move from the tissues to the lungs (Fahlman et al., 2009). Elevated ${\overset{\cdot }{\text{Q}}}_{\text{tot}}$ at this stage of the dive would result in a decrease in the ${\overset{\cdot }{V}}_{A}$/${\overset{\cdot }{\text{Q}}}_{\text{tot}}$ ratio, which would enhance exchange and removal of N_2_. It has been hypothesized that disturbance of normal diving homeostasis, such as exposure to man-made sound, capture in nets, or any stressful situation may alter the physiology and result in conditions that enhance N_2_ uptake and the risk for gas emboli (Fernandez et al., 2005; Hooker et al., 2009; Fahlman et al., 2014; García-Párraga et al., 2014). In turtles, it has been suggested that the stress associated with incidental capture in fishing gear result in a sympathetic response, which opens the arterial pulmonary sphincter, increases pulmonary blood flow, decreases the${\overset{\cdot }{V}}_{A}$/${\overset{\cdot }{\text{Q}}}_{\text{tot}}$ ratio and increase N_2_ uptake and risk of gas emboli (García-Párraga et al., 2014, 2017; Fahlman et al., 2017a). In cetaceans, active management of the pulmonary perfusion has been hypothesized as a mechanism that allow the cetaceans to vary the level of the ${\overset{\cdot }{V}}_{A}$/${\overset{\cdot }{\text{Q}}}_{\text{tot}}$ match in the lung, and develop a shunt even at shallow depths that does not depend on hydrostatic compression (Garcia Párraga et al., 2018). Similar to the turtles, stress in cetaceans may also alter perfusion and the${\overset{\cdot }{V}}_{A}$/${\overset{\cdot }{\text{Q}}}_{\text{tot}}$ relationship causing increased pulmonary blood flow, increased uptake of N_2_ and risk of gas emboli (Fahlman et al., 2006; Hooker et al., 2009). Theoretical modeling has indicated that stress related changes in ${\overset{\cdot }{\text{Q}}}_{\text{tot}}$ may increase the risk for gas emboli, and empirical data in the narwhal indicate that the large changes in the diving heart rate occurs deeper under stressful situations (around 170 m) as compared with natural dives (Fahlman et al., 2009, 2014; Hooker et al., 2009; Williams et al., 2017). Thus, this reversal of the diving bradycardia may result in elevated pulmonary blood flow, changes in blood flow distribution within the lung and changes in ${\overset{\cdot }{V}}_{A}$/${\overset{\cdot }{\text{Q}}}_{\text{tot}}$, resulting in elevated N_2_ uptake and risk for gas emboli (Garcia Párraga et al., 2018).

In summary, in the current study we show that the dive behavior in deep diving bottlenose dolphins likely requires morphological differences resulting in a greater O_2_ storage capacity as compared with the coastal ecotype. It is also possible that the foraging behavior of the offshore population, with longer periods of gliding, results in lower foraging metabolic costs. Our results also indicate the importance of species-specific data when predicting physiological responses of animals. By updating the compliance estimates for the respiratory system, we show additional evidence of how variation in the ventilation/perfusion relationship is able to alter exchange of gases. This result provides additional support for our hypothesis of how marine mammals manage gases during diving and how stress may alter physiology and cause increased N_2_ uptake and risk of gas emboli from forming.

## Data availability

The theoretical model and data used in this paper are made freely available at: osf.io/eyxpa.

## Author contributions

AF helped collect the dive data, performed the modeling work, data analysis, wrote the first draft of the paper. FHJ collected and extracted the dive data, and helped edit the paper. PT provided the tools to collect the dive data, and helped edit the paper. RW helped collect the dive data and edited the paper.

### Conflict of interest statement

The authors declare that the research was conducted in the absence of any commercial or financial relationships that could be construed as a potential conflict of interest.
